# Interleukin 6 Deficiency Modulates the Hypothalamic Expression of Energy Balance Regulating Peptides during Pregnancy in Mice

**DOI:** 10.1371/journal.pone.0072339

**Published:** 2013-08-28

**Authors:** Patricia Pazos, Luis Lima, Felipe F. Casanueva, Carlos Diéguez, María C. García

**Affiliations:** 1 Department of Physiology/Research Center of Molecular Medicine and Chronic Diseases (CIMUS), University of Santiago de Compostela, Santiago de Compostela, Spain; 2 Instituto de Investigación Sanitaria de Santiago de Compostela, Santiago de Compostela, Spain; 3 CIBER Fisiopatología Obesidad y Nutrición (CB06/03), Instituto de Salud Carlos III (ISCIII), Ministerio de Economía y Competitividad (MINECO), Santiago de Compostela, Spain; 4 Laboratorio de Endocrinología Molecular y Celular, Complexo Hospitalario Universitario de Santiago (CHUS), Santiago de Compostela, Spain; University of Cordoba, Spain

## Abstract

Pregnancy is associated with hyperphagia, increased adiposity and multiple neuroendocrine adaptations. Maternal adipose tissue secretes rising amounts of interleukin 6 (IL6), which acts peripherally modulating metabolic function and centrally increasing energy expenditure and reducing body fat. To explore the role of IL6 in the central mechanisms governing dam's energy homeostasis, early, mid and late pregnant (gestational days 7, 13 and 18) wild-type (WT) and *Il6* knockout mice (*Il6-*KO) were compared with virgin controls at diestrus. Food intake, body weight and composition as well as indirect calorimetry measurements were performed *in vivo*. Anabolic and orexigenic peptides: neuropeptide Y (*Npy*) and agouti-related peptide (*Agrp*); and catabolic and anorectic neuropeptides: proopiomelanocortin (*Pomc*), corticotrophin and thyrotropin-releasing hormone (*Crh* and *Trh*) mRNA levels were determined by in situ hybridization. Real time-PCR and western-blot were used for additional tissue gene expression and protein studies. Non-pregnant *Il6*-KO mice were leaner than WT mice due to a decrease in fat but not in lean body mass. Pregnant *Il6*-KO mice had higher fat accretion despite similar body weight gain than WT controls. A decreased fat utilization in absence of *Il6* might explain this effect, as shown by increased respiratory exchange ratio (RER) in virgin *Il6*-KO mice. *Il6* mRNA levels were markedly enhanced in adipose tissue but reduced in hypothalamus of mid and late pregnant WT mice. *Trh* expression was also stimulated at gestational day 13 and lack of *Il6* blunted this effect. Conversely, in late pregnant mice lessened hypothalamic *Il6 receptor alpha (Il6ra)*, *Pomc* and *Crh* mRNA were observed. *Il6* deficiency during this stage up-regulated *Npy* and *Agrp* expression, while restoring *Pomc* mRNA levels to virgin values. Together these results demonstrate that IL6/IL6Ra system modulates *Npy*/*Agrp*, *Pomc* and *Trh* expression during mouse pregnancy, supporting a role of IL6 in the central regulation of body fat in this physiological state.

## Introduction

Energy balance is largely regulated by the central nervous system (CNS) via a homeostatic system. Central regulatory networks, located mainly in the hypothalamus and brainstem, sense metabolic status from widely divergent afferent signals (hormones, nutrients and neural signals) and modify the expression and release of specific neurotransmitters/neuromodulators with potent effects on energy intake and expenditure [1], [2]. Disturbances in the activity of these central circuitries and/or peripheral signaling pathways may lead to changes in feeding behavior, substrate metabolism and energy expenditure; ultimately favoring an adjustment of body weight and body composition [3].

Afferent endocrine signals encoding energy status may arise from different sites including: i) fat, which secretes adipokines such as leptin, adiponectin, resistin and interleukin-6 (IL6), ii) pancreas, which produces insulin and iii) gastrointestinal organs, which release hormones such as ghrelin, glucagon-like peptide I and peptide YY [1], [4], [5]. Within the hypothalamus, two functionally opposing subpopulations of neurons in the arcuate nucleus (ARC) represent a key integrative site for these signals. One subpopulation produces gamma aminobutyric-acid (GABA) and the orexigenic neuropeptideY (NPY) and agouti-related peptide (AGRP). The other produces cocaine and amphetamine regulated transcript (CART) and proopiomelanocortin (POMC)-derived peptides, such as alpha melanocyte stimulating hormone (αMSH), that promote anorexia by inhibiting food intake and increasing catabolic processes. αMSH modulate its downstream homeostatic signaling via their action at melanocortin receptors MC3R and MC4R, which are antagonized by AGRP [3]. Additionally, AGRP/NPY neurons block the αMSH effects by activating NPY receptors (Y1-Y5) on the MC4-R bearing cells and through direct and indirect GABA-ergic inhibitory inputs [6].

Both groups of cells project to several brain areas such as the paraventricular nucleus (PVN) and lateral hypothalamic area (LHA). The major neurotransmitters released from the PVN include: oxytocin, arginine-vasopressin (AVP), corticotrophin and thyrotropin-releasing hormone (CRH and TRH) [7]. CRH and TRH have rather overlapping roles in the control of energy balance. Thus, central administration of both neuropeptides decreases food intake and increases locomotor activity, oxygen consumption and brown adipose tissue thermogenesis. These effects maybe exerted directly at the CNS, modulating autonomic function, or via the hypothalamic-pituitary adrenal and thyroid axes (HPA and HPT), inducing glucocorticoid and thyroid hormone release [7]–[10].

IL6 is a pleiotropic immunomodulatory cytokine produced not only by the cells of immune system but also by cells in neuroendocrine and endocrine tissues, such as those in the hypothalamus, adipose tissue, skeletal muscle and reproductive organs [11]. Over the last decade this cytokine has attracted particular attention because of its regulatory role on peripheral lipid metabolism [12]–[14] and contradictory multi-systemic effects on insulin action [15]–[19]. Growing evidence suggest that centrally acting IL6 could also have a physiological role in the regulation of food intake, energy expenditure and adiposity. Obesity, exercise and diabetes are associated with elevated plasma IL6 concentrations while cerebrospinal fluid (CSF) IL6 levels remain unchanged [20], [21]. However, IL6 is found in the CNS in health and disease, with cellular sources being glial cells and neurons [22], and might regulate the hypothalamic circuits involved in energy homeostasis in a gender and age dependent manner. Intracerebroventricular, but not peripheral IL6 administration, increases energy expenditure and reduces food intake and weight of visceral fat depots [14], [23]. In fact, transgenic female mice with CNS-restricted *Il6* over-expression show decreased *Npy* and *Agrp* mRNA levels at all ages, and a late increase in *Pomc* expression in the arcuate nucleus (ARC) [24]. Conversely, *Il6* deficiency in mice decreases fat oxidation [13], [25], [26] and leads to a mature onset obese phenotype [14], which is accompanied by reduced expression of energy balance regulating peptides in the PVN [27]. The IL6 receptor alpha (IL6Ra), crucial for ligand binding, has been shown to be abundantly produced in the PVN and co-expressed to a high extent with CRH, AVP, oxytocin and TRH suggesting that IL6 could stimulate the expression of these peptides directly [27].

During pregnancy adjustments are made to mother's homeostatic mechanisms that regulate food intake and the metabolism of nutrients [28], [29]. Changes in lipid metabolism result in accumulation of maternal fat stores in early and mid-pregnancy, and enhance fat mobilization in late pregnancy. In the early stage, promotion of lipogenesis and reduced lipolysis are mediated by increased insulin sensitivity and secretion as well as progesterone levels. This anabolic hormonal milieu is reinforced by the development of maternal hyperphagia, which is sustained throughout pregnancy [30], [31]. As pregnancy proceeds, insulin sensitivity is impaired and lipolysis and fat oxidation are enhanced, allowing the use of lipids as maternal energy source [32]. In this context, a progressive increase in adipose tissue production and serum levels of different adipokines as leptin, resistin and IL6 has been reported in both rodent and human pregnancies [33]–[37]. In the pregnant rat, circulating IL6 levels are enhanced during the entire gestational period and paralleled its CSF concentration during early and mid-stages but, similarly to leptin CSF levels, decreased to virgin control values in late pregnancy [35]. At this gestational time the ARC neural pathways influencing food consumption are activated, leading to an increased *Npy* and *Agrp* expression and a decline in *Pomc* mRNA content [31], [38]. Moreover throughout most of pregnancy, but also in lactation, the basal activity of the HPA axis is inhibited, predominantly reflecting a reduced drive by CRH and AVP neurons in the PVN [39]. However, despite these circumstantial evidences, the relative importance of IL6 as a central regulator of food intake and adiposity during pregnancy remains to be demonstrated. Thus, in the present study we sought to determine whether pregnancy-related changes in ARC and PVN circuitries involved in energy homeostasis are affected by knockout of *Il6* in mice (*Il6*-KO). We also investigated whether the putative effects of endogenous IL6 could be mediated by changes in hypothalamic *Il6ra* expression during this physiological state.

## Materials and Methods

### Animals

B6.129S2-IL6 tm1kopf/J (*Il6*-KO) [40], that had undergone eleven backcrosses to the C57BL/6 background and their congenic wild-type controls C57BL/6J mice (WT) were purchased from Jackson laboratories (Charles River, Barcelona, Spain; stock numbers 002650 and 000664, respectively). Since the *Il6* mutation is maintained in the C57BL/6J 000664 background, WT and *Il6*-KO mice colonies were bred on homozygous conditions and maintained in the same room of the Santiago de Compostela Animal house under specific pathogen-free conditions (SPF). The absence of full-length *Il6* transcripts in *Il6*-KO mice was confirmed by RT-PCR. After weaning, animals were housed 5–6 per cage under controlled temperature conditions (22°C) and a 12 hour light-dark cycle, with free access to water and rodent chow (2019 s, Teklad Global, Harlan, Spain). Age matched female WT and *Il6*-KO mice (12–15 weeks old) were always used and allocated to either virgin (unmated) or pregnant groups prior to the start of the experiments. To obtain pregnant animals, female *Il6*-KO and WT mice were mated with stud mice of the same genotype and timed pregnant from the day the vaginal plug was detected (day 0 post coitum). Ethical approval was obtained from the University of Santiago de Compostela Bio-ethics Committee (ID: PX06/208067) and all the procedures were conducted according to the regulations of the European Community.

### Experimental design

In a first experiment, food intake and body weight were monitored daily for 18 days in single housed virgin and pregnant, 12 weeks old WT and *Il6*-KO mice (n = 15–16 animals/pregnant group and n = 21 animals/virgin group). Body length was measured on an independent group of sedated virgin WT and *Il6*-KO mice with a ruler from nose to anus (n = 6 animals/group). At the end of study period half of the pregnant mice were either sacrificed (n = 8 animals/group) or allowed to give birth naturally (n = 7–8/group). Combined weight of placentae and fetuses as well as newborn mice body weight were measured.

In a second study, longitudinal measurements of whole-fat content were carried out in groups of 15 weeks old unmated and mated mice at gestational days 7, 13 and 18 (corresponding to early, mid and late pregnancy, respectively) (n = 9–10 animals/pregnant group n = 5–7 animals/virgin group) using a nuclear magnetic resonance (NMR) spectroscopy device EchoMRI-700™ (Echo Medical Systems, Houston, TX). Transversal analysis of specific fat depots and sample collection for subsequent expression studies by real-time quantitative RT-PCR were performed in 12 weeks old mice. Animals were time pregnant and on the corresponding date of pregnancy were anesthetized, and after collection of serum samples by cardiac puncture, were sacrificed by decapitation. Age and genotype matched virgin females, identified as diestrus by vaginal cytology, were used as controls (gestational days 7 (n = 15/pregnant group and n = 14–16/virgin group), 13 (n = 18–20/pregnant group and n = 18–21/virgin group) or 18 (n = 22–24/pregnant group and n = 24–25/virgin group). Dissection and weighing of intra-abdominal (mesenteric, gonadal and retroperitoneal) and subcutaneous fat depots was performed as previously described [41]. Abdominal fat distribution was determined as percentage of the combined weight of the dissected depots from non pregnant controls of the same genotype. Gonadal and brown adipose tissue samples (BAT), complete brains or whole hypothalami were removed, quickly frozen on dry ice and stored at –80°C prior to analysis of *Il6*, *Il6ra* or neuropeptide expression.

In a third experiment energy expenditure and respiratory exchange ratio (RER = VCO2/VO2) were measured in 12 weeks old virgin WT and *Il6*-KO mice. Animals were monitored in a 12-cage indirect calorimetry, food intake and locomotor activity monitoring system (TSE LabMaster, TSE Systems, Bad Homburg, Germany) as previously described [42]. Mice were acclimated for 48 hr to the test chambers and then were monitored for an additional 24 hr. Measurements were taken every 40 minutes. Data collected from the last 24 hr were used to calculate energy expenditure and average RER during 12 h-light and 12 h-dark phases of the daily cycle.

### Serum assays

Circulating serum levels of C-reactive protein (CRP), leptin and interleukin 6 were assayed by ELISA, using commercial kits (CRP, Life Diagnostics, Knypersley, UK; Leptin, Crystal Chem, Downers Grove, IL, USA and IL6, Abcam, Cambridge, UK) according to the manufacturer's instructions.

### In situ hybridization

Serial 16 µm-thick coronal sections were cut on a cryostat at −20°C, mounted onto polylysine-coated slides (Thermo Scientific, Spain) and immediately stored at −80°C. *Npy*, *Agrp*, *Pomc*, *Trh* and *Crh* mRNA levels were determined using 42-45-mer specific antisense oligo-probes (Table S1 in File S1). The probes were 3′-end labeled with ^35^S-αdATP (1250 Ci/mmol; Perkin-Elmer, Boston, USA) and terminal deoxynucleotidyl transferase (New England BioLabs, UK). Specificity of the hybridization signal was confirmed by performing co-hybridization studies with an excess of unlabeled probes (not shown). In situ hybridization was carried out as previously described [38]. Tissue sections were fixed with 4% paraformaldehyde in 0.1 M phosphate buffer (pH 7.4) at room temperature for 30 min, dehydrated through 70, 80, 90, 95% and absolute ethanol (5 min each). Sections were removed from ethanol, allowed to dry and hybridized overnight in a humid chamber at 42°C, in a hybridization solution containing 4× SSC, 50% deionized formamide, 1× Denhardt's solution, 10 µg/mL sheared single-stranded salmon sperm DNA, 10% dextran sulfate, DTT 50 mM and 0.5–1×10^6^ cpm/slide of the labeled probes. After hybridization, sections were rinsed in 1xSSC at room temperature, sequentially washed in 1xSSC at 55°C (30 min/wash, 4 washes in total) and room temperature for 1 h, and finally rinsed in 70% ethanol with 300 mM ammonium acetate. After air-dried hybridized sections were apposed to autoradiography film (Kodak Biomax MR) at room temperature for 5 days (*Npy*, *Agrp*, *Pomc*, *Trh* mRNA) and 7 days (*Crh* mRNA).

To compare anatomically similar regions, slides were matched according to the mouse atlas of Franklin and Paxinos [43]. In each experiment with a specific transcript the slides (3–4 slides per animal/4 sections in each slide) from at least three to four control virgin and pregnant mice, at each gestational age, were processed together and were always exposed to the same autoradiographic film. Autorradiographic images were scanned and the optical density of the specific hybridization signal was quantified using the image analysis software ImageJ 1.40 (National Institutes of Health, USA).

### Real time qPCR

Real time quantitative RT-PCR (qPCR) analysis was carried out as previously described [44]. Total RNA was extracted from frozen abdominal white adipose tissue (WAT), hypothalami or brown adipose tissue (BAT) using TRIzol according to the manufacturer's instructions (Invitrogen, Barcelona, Spain). Complementary DNAs were synthesized from 500 ng of total RNA in a 30 μl reaction using 200 U Maloney murine leukemia virus reverse transcriptase and random hexamer primers (Invitrogen, Barcelona, Spain). Quantitative real time PCR was performed using an ABI PRISM 7300HT Sequence Detection System (Applied Biosystems; Foster City, CA, USA), with 2 μl aliquots of the resulting cDNAs and specific Taqman qRT-PCR primers and probes for *Il6*, *Il6ra*, *Ucp1* and *Ucp3* (Table S1 in File S1). Amplification of *18S rRNA* (*Rn18S*) was performed at the same time to normalize the level of mRNA expression. A non template reaction was included during each experiment to control for DNA contamination. The PCR cycling conditions included an initial denaturation at 50°C for 10 min, followed by 40 cycles at 95°C for 15 s and 60°C for 1 min. A standard curve was run in each assay, with an arbitrary value assigned to the highest standard and corresponding values to the subsequent dilutions. Each cDNA sample was run in duplicate. The relative abundance of *Il6*, *Il6ra, Ucp1* and *Ucp3* targets were normalized against that of *Rn18s* and expressed in percentage respect to the average value of the WT virgin control group.

### Western-blot

Western-blots were performed as previously described [44]. Briefly, total protein lysates from BAT (20 µg) samples were subjected to SDS-PAGE, electrotransferred onto a polyvinylidene difluoride membrane (Immobilon-P, Millipore, Billerica, MA, USA) and probed with antibodies against UCP1 (ab10983), UCP3 (ab3477, Abcam, Cambridge, UK) and alpha-tubulin (T5168, Sigma-Aldrich, St. Louis, MO, USA). The antibodies dilution was 1∶1000. For protein detection biotinylated secondary antibody conjugates, diluted (1∶5000) and chemiluminescence (Thermo Fisher Scientific, Madrid, Spain) were used. Protein levels were normalized to alpha-tubulin for each sample and expressed in percentage respect to the average value of the WT virgin control group.

### Oil-Red staining

Frozen BAT samples preserved in OCT were cryosectioned into 10 µM sections, mounted onto microscope slides and stored at −10°C until staining. Slides were allowed to acclimate to room temperature for approximately 10–15 min prior to staining. Tissue was then fixed in 10% neutral buffered formalin for 5 min and briefly washed in water. The slides were then rinsed in 60% isopropanol and placed into freshly prepared Oil Red O working solution (Sigma-Aldrich, St. Louis, MO, USA). Slides were allowed to stain for 15 min at room temperature. After staining, slides were rinsed with 60% isopropanol and the nuclei were lightly stained with hematoxylin stain (Sigma-Aldrich, St. Louis, MO, USA). Lastly, the slides were washed 3 times in water and coverslips were applied using aqueous mounting media. The slides were then visualized on a light microscope at 10× magnification.

### Statistical analysis

Results are given as means ± SEM. All data were analyzed using Graph Pad Prism version 6.00 for Windows (GraphPad Software, La Jolla, California USA). Comparisons between two groups were performed with unpaired two-tailed Student's t test and one-way analysis of variance (ANOVA), followed by the Bonferroni post hoc test, when differences between more than two experimental groups were analyzed. Data derived from the same animals at several times were analyzed with two-way ANOVA for repeated measurements to evaluate differences between experimental groups. P<0.05 was considered statistically significant.

## Results

### Il6-KO mice show similar weight gain and fat mass but higher fat accretion than WT mice during pregnancy

**Figure 1:**
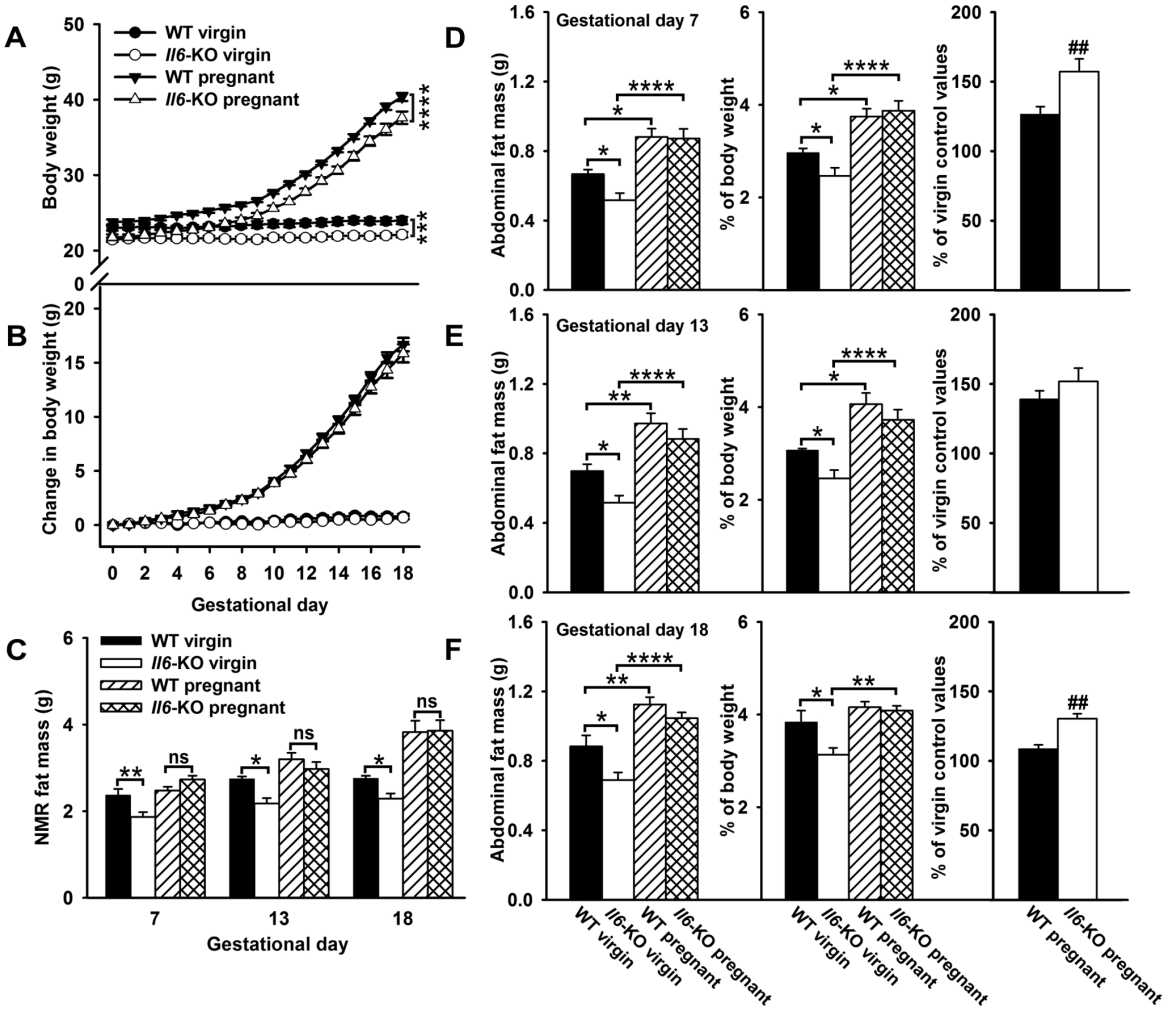
Normal body weight gain, fat mass but higher abdominal fat accretion in pregnant *Il6*-KO mice. **A–B**. Body weight (A) and change in body mass (B) were monitored daily in 12 weeks old mated (n = 15–16) and unmated WT and *Il6*-KO mice (n = 21). **C**. Longitudinal measurements of NMR fat were performed in 15 weeks old mice at gestational days 7, 13 and 18 (virgin, n = 5–7 and pregnan, t n = 9–10). **D–F**. For transversal analysis of absolute (right panel) and relative dissected abdominal fat mass (middle and left panel), independent groups of 12 weeks old time-pregnant mice were sacrificed on gestational days 7 (D, n = 8–9), 13 (E, n = 8–9) and 18 (F, n = 11). Age and genotype matched virgin females were used as controls (n = 7–11). Relative fat mass values were calculated in percentage to maternal body weight (middle panel), excluding the contribution of placentae and fetuses, and normalized to virgin control values of each genotype (F). Data are expressed as mean ± SEM. One-way ANOVA and two-way ANOVA for repeated measurements, *P<0.05, **P<0.01,***P <0.001 and ****P<0.0001, ns =  not significant; two-tailed t-test ##*P*<0.01 versus corresponding WT pregnant controls.

Firstly, we studied the effect of lack of endogenous *Il6* in the pregnancy-related changes on body weight (Figure 1A). The mean body weight of 12 weeks-old *Il6*-KO female mice used in this study, unmated or mated, was lower than that of WT mice (average body weight at day 0 of pregnancy: WT mice  = 23.36±0.24 g versus *Il6*-KO mice  = 21.56±0.23 g, P<0.001, t-test). There were no differences in their longitudinal length at this age between genotypes (WT mice  = 8.833±0.06 cm versus *Il6*-KO mice  = 8.816±0.075 cm). Maternal body weight increased exponentially throughout pregnancy, but remained a 7% lower in the *Il6*-KO pregnant than in WT pregnant group until gestational day 18 (genotype F (1, 29)  = 28.41, time F (18, 522)  = 1002, P<0.0001 and genotype X time interaction F (18, 522)  = 0.8435, not significant, two-way repeated measures ANOVA). A similar genotype effect was observed on the weight of non pregnant groups (genotype F (1, 40)  = 14.75, time F (18, 720)  = 18.74 and genotype X time interaction F (18, 720)  = 2.326, P<0.001, P<0.0001 and P<0.001, respectively). However, the body weight gain was similar in WT and *Il6*-KO mice whether pregnant or not in all time points studied, with values for virgin groups at the end of the 18 days-study period of: WT mice  = 1.024±0.242 g versus *Il6*-KO mice  = 0.676±0.150; and pregnant groups of: WT mice  = 16.672±0.619 g versus *Il6*-KO mice  = 15.854±0.835 g (Figure 1B). The mean number and weight of embryos and placentae in uteri of 18-days pregnant mice were also similar between genotypes, as were the litter sizes and the weight of newborn mice at birth (Table S2 in File S2).

Taking into account that expansion of fatty tissue, besides conceptus growth, is the main contributor to gestational weight gain [30], [32], we therefore evaluated body fat mass by NMR in 15 weeks old mice WT and *Il6*-KO mice throughout pregnancy. As expected [26] in 15 weeks old non-pregnant adult, *Il6*-KO mice had a lower total fat mass content than WT mice during the NMR study (−18% as a mean, genotype F (1, 10)  = 18.26, time F (2, 20)  = 11.06, P<0.01 and genotype X time F (2, 20)  = 0.107, P = 0.899, two-way repeated measures ANOVA) (Figure 1C). As a significant overall effect of genotype was detected, a Bonferroni post-test was applied for the pairwise comparison (e.g. fat mass of virgin WT  = 2.366±0.117 g vs fat mass of virgin *Il6*-KO at day 7 = 1.866±0.095 g). The results for genotype effect were significant at day 7, 13 and 18 (P<0.01, P<0.05 and P<0.05, respectively). No effect of *Il6* deficiency was seen in lean body mass values, which were similar in virgin WT and *Il6*-KO mice at the beginning (WT mice  = 17.505±0.435 g versus *Il6*-KO mice  = 16.634±0.501 g) and at the end of the experimental period (WT mice  = 18.252±0.465 g versus *Il6*-KO mice  = 17.444±0.382 g) (Figure S1A in File S3). On the contrary, pregnancy in all its phases is able to revert the effect of *Il6* deficiency on total adipose tissue levels (genotype F (1, 18)  = 0.0007, P = 0.978 and time F (2, 36)  = 38.82, P < 0.0001). Nevertheless, we did observe a trend to lower lean body mass values in *Il6*-KO pregnant mice in relation to their WT controls, which reached statistical significance only in late pregnant animals when expressed in absolute (Figure S1A in File S3) but not relative to body weight values (Figure S1B in File S3).

Since *Il6*-KO mice appeared to have higher fat mass accretion than WT mice during pregnancy, dissection of specific fat depots in independent groups of 12 weeks old animals was performed at gestational days 7, 13 and 18 (early, mid and late pregnant, respectively). Thus, confirming the NMR data, the absolute and relative weights of visceral fat pads in all groups of non-pregnant *Il6*-KO mice were lower than those of corresponding WT virgin controls (on average: −22, −23 and −20%, P<0.05, one-way ANOVA) (Figure 1D, E and F, left and middle panel). Both parameters were similar in pregnant mice of both genotypes at each stage of gestation. As expected, early, mid and late pregnant WT and *Il6*-KO mice also exhibited an increased absolute visceral fat, compared to virgin controls of each genotype (P<0.05, P<0.01 and P<0.01, respectively) (Figure 1D, E and F, left panel). However, when these values are corrected by body weight, the differences between pregnant and virgin mice of each genotype were significant only at early and mid, but not at late pregnancy (P<0.05 for all) (Figure 1D, E and F, middle panel). At gestational day 18, pregnant *Il6*-KO mice showed higher levels of relative fat mass than corresponding virgin controls, with no differences between WT mice groups (P<0.01) (Figure 1F, middle panel). Analysis of relative abdominal fat mass content in relation to non-pregnant mice values in each genotype revealed that, early and late-pregnant *Il6*-KO mice showed 30 and 16% higher fat accretion than WT mice (P<0.01 for early and late-pregnant groups by t-test) (Figure 1D and F, left panel). A similar effect of lack of *Il6* on fat accretion was observed in subcutaneous adipose tissue of early and mid-pregnant mice (Figure S2A and B in File S4).

Additionally, serum leptin levels were also quantified in samples from virgin and late pregnant WT and *Il6*-KO mice. As previously described [45], [46], by the end of pregnancy serum leptin levels were markedly higher than those in non-pregnant mice in both genotypes (P<0.001, one-way ANOVA) (Figure S3 in File S5). In agreement with the fat mass results, virgin but not pregnant *Il6*-KO mice showed significantly lower serum leptin concentrations that their correspondent WT controls (P<0.05).

### Lack of Il6 does not affect relative food intake during pregnancy

**Figure 2:**
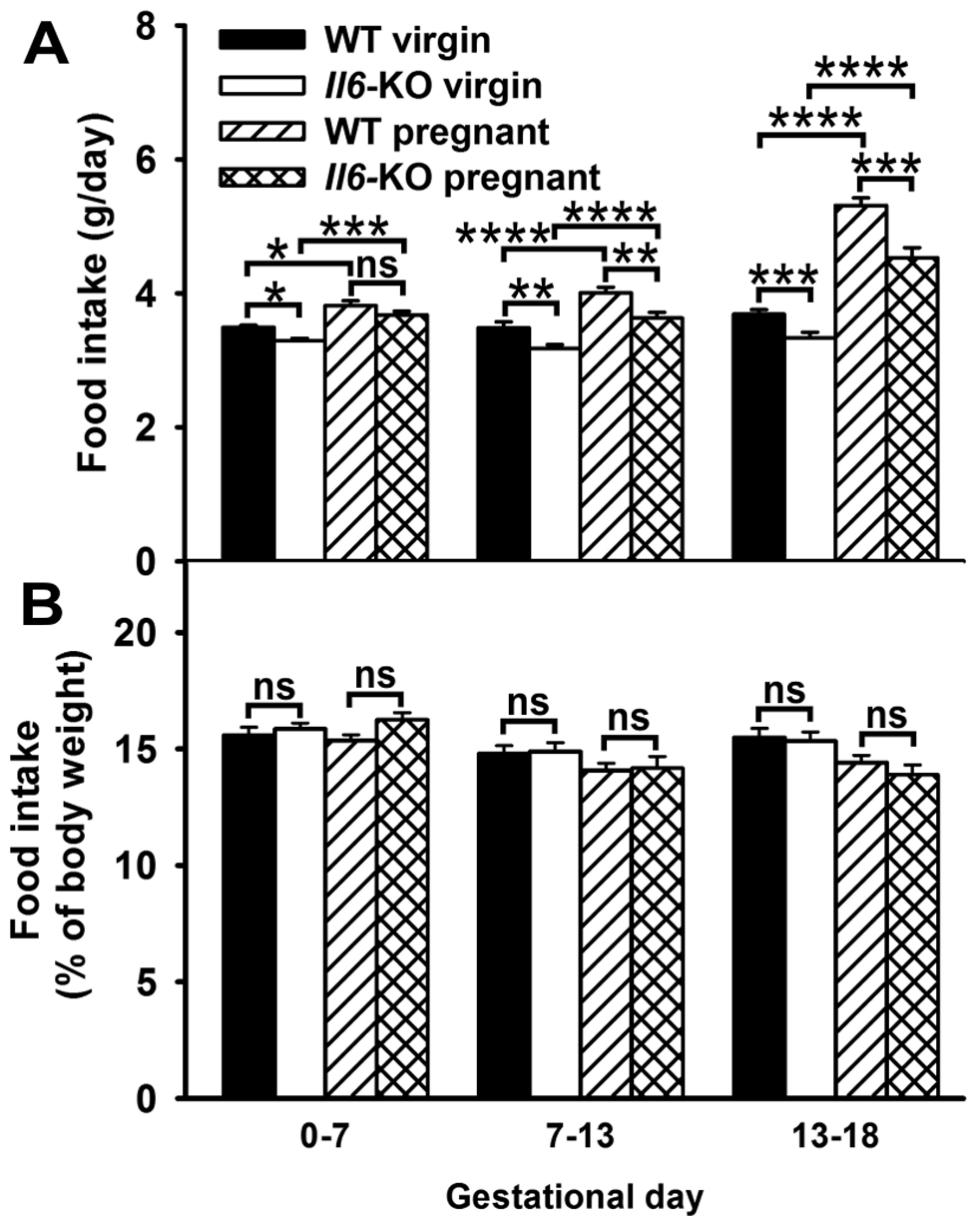
Average daily intake in *Il6*-KO mice throughout pregnancy. Food intake was measured daily in individually housed 12 weeks old virgin (n = 21) and pregnant WT and *Il6*-KO mice (n = 15–16). **A–B**. Average daily intake (A) was calculated for early (gestational days 0–7), mid (gestational days 7–13) and late pregnancy (gestational days 13–18). Food intake values normalized in percentage to animal body weight (B). Two-way ANOVA for repeated measurements, *P<0.05, **P<0.01, ***P <0.001 and ****P<0.0001, ns =  not significant.

Next, we evaluated whether the profile of fat accretion in *Il6*-KO pregnant mice was related to changes in average daily food intake during early (gestational days 0–7), mid (gestational days 7–13) and late gestation (gestational days 13–18) (Figure 2). Non-pregnant 12 weeks old *Il6*-KO mice ate less amount of food than their corresponding WT controls when values are expressed on an absolute (−7,3% as a mean, Figure 2A), but not on a weight corrected basis (Figure 2B) (absolute: genotype F (1, 40)  = 17.86, P < 0.001, time F (2, 80)  = 5.823, P < 0,01 and genotype X time F (2, 80)  = 1.180, not significant, two-way repeated measures ANOVA), (relative: time F (2, 80)  = 5.581, P<0.01, genotype F (1, 40)  = 0.0246 and genotype X time F (2, 80)  = 0.322, not significant). Food consumption increased progressively during pregnancy (group F (3, 69)  = 55.61, time F (2, 138)  = 81.32 and group X time F (6, 138)  = 15.13, P < 0.0001 for all), and was higher than non-pregnant levels by its first week (P<0.05, Figure 2A). At this time point absolute food intake values were similar in both genotypes, being lower in *Il6*-KO than in WT mice throughout the rest of the gestational period (genotype F (1, 29)  = 15.33, P < 0.001, time F (2, 58)  = 125.7, P < 0.001 and genotype X time F (2, 58)  = 4.324, P<0.05) (gestational days 13 and 18: P<0.01 and P<0.001, respectively). However, when food intake values in pregnant mice were corrected by body mass no effect of *Il6* deficiency was seen in this parameter (Figure 2B) (genotype F (1, 29)  = 0.151, P = 0.443, time F (2, 58)  = 25.521, P < 0.0001 and genotype X time F (2, 58)  = 3.367, P<0.05).

### Lack of Il6 increases respiratory exchange ratio in virgin mice

**Figure 3:**
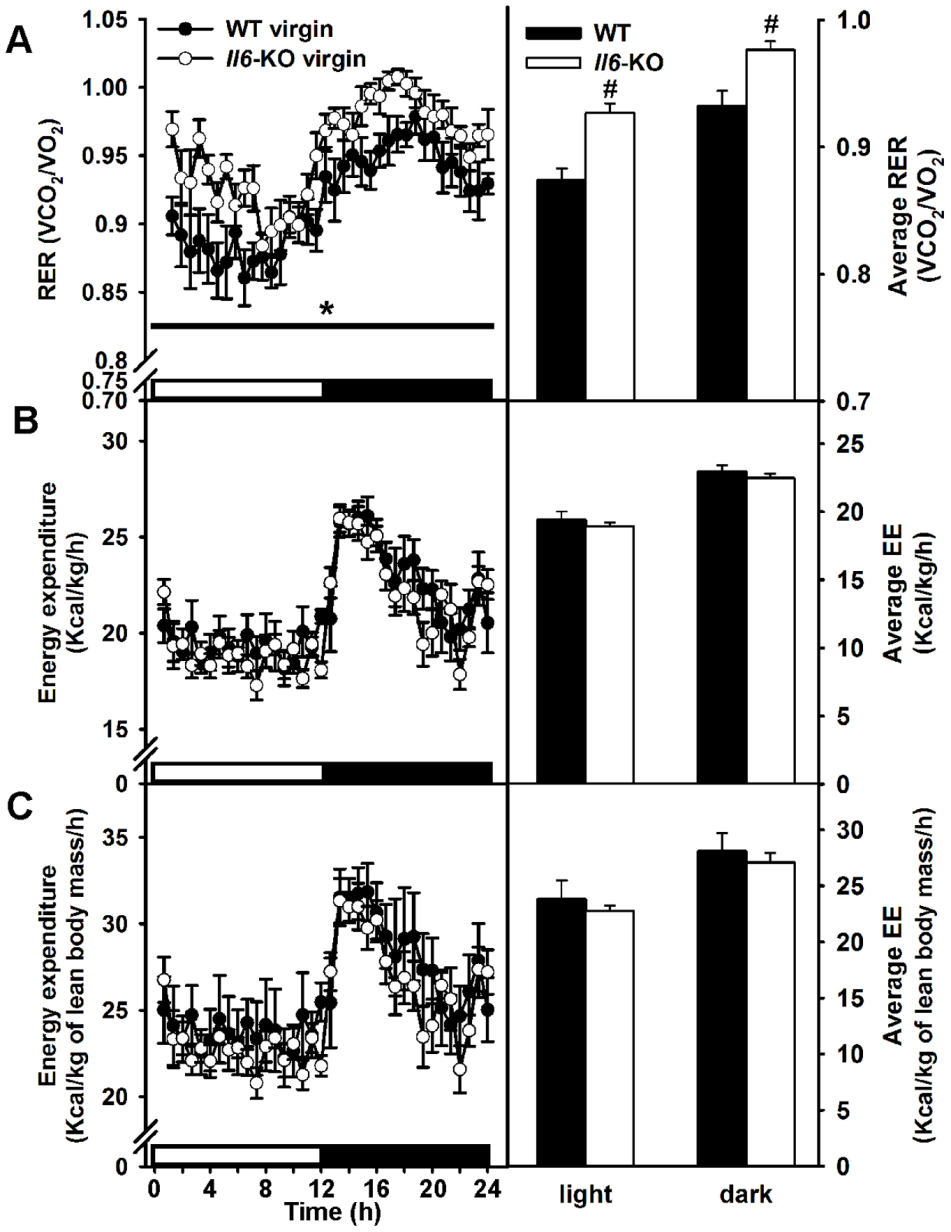
Increased respiratory exchange ratio in *Il6*-KO mice. **A–C**. Respiratory exchange ratio (RER) (A), energy expenditure corrected by body weight (B) and lean mass (C) were measured in young (12 weeks old) WT and *Il6*-KO female mice at room temperature. Black horizontal bars depict the dark period in a 12:12-h light-dark cycle. Data were collected during a 24-h period (8:00 a.m. till 8:00 a.m.). Average values of RER as well as body weight and lean mass corrected energy expenditure are shown in right panels. Values are expressed as means ± SEM, n = 6. Two-way ANOVA for repeated measurements, *P < 0.05 and two-tailed t-test, #P<0.05 for *Il6*-KO versus WT mice.

In order to ascertain whether changes in energy expenditure or metabolic substrate preference (RER) in *Il6*-KO mice could induce the subsequent fat accumulation during pregnancy, both parameters were analyzed by indirect calorimetry. To overcome the contribution of fetal lean body mass to the analysis, 12 weeks old virgin WT and *Il6*-KO mice were used in this study. *Il6*-KO mice showed an increased RER in comparison with WT mice (Figure 3A) (genotype F (1,10)  = 6.268, P<0.05, time F (35,350)  = 1464 and genotype X time interaction F (35, 350)  = 1,206, not significant, two-way repeated measures ANOVA) during both the light and dark phases of the circadian cycle (P<0.05) (Figure 3A, right panel), suggesting that a low lipid-to-carbohydrate oxidation could contribute to normalize its fat stores during pregnancy. No significant differences in total energy expenditure, when corrected by body weight (Figure 3B) or lean body mass (Figure 3C), were detected. On the contrary, indications of an altered BAT thermogenic capacity of *Il6*-KO pregnant mice were observed, suggesting that lack of *Il6* might cause a decrease in energy expenditure during pregnancy. Therefore, lower *Ucp1* and *Ucp3* mRNA levels (Figure S4A and B in File S6) and increased lipid content (Figure S4E in File S6), as measured by increased oil-red staining, were found in BAT of mid-pregnant *Il6*-KO in comparison to WT pregnant controls. However, these observations were confirmed by western blot studies in the case of UCP3 but not UCP1 (Figure S4C and D in File S6).

### Increased serum IL6 and CRP levels during pregnancy in WT mice

**Figure 4:**
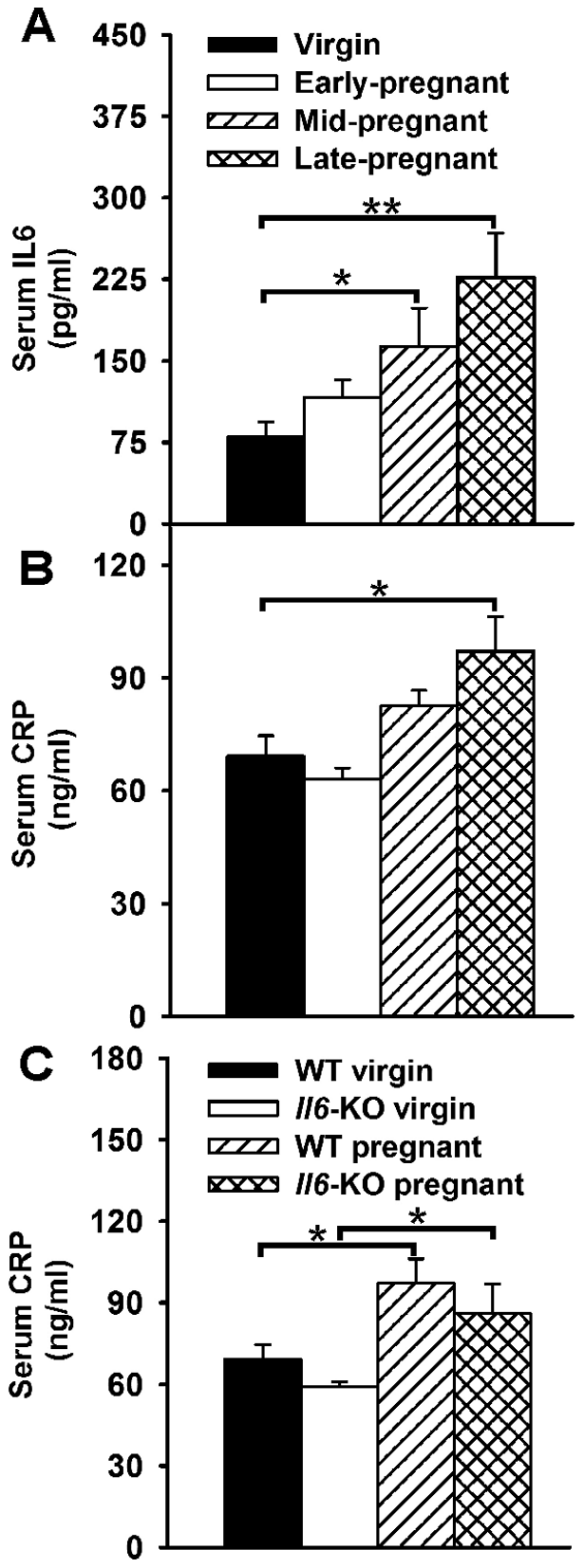
Serum IL6 and CRP levels during pregnancy in WT and *Il6*-KO mice. **A–B**. Serum IL6 (A) and CRP (B) levels as assessed by ELISA in virgin and pregnant WT mice at gestational days 7, 13 and 18 (n = 6–7). **D**. Serum CRP levels in virgin and late pregnant WT and *Il6*-KO mice. Data are expressed as mean ± SEM. One-way ANOVA, *P<0.05, **P<0.01.

Circulating levels of IL6 and CRP were also assessed during pregnancy. As expected [37], serum IL6 levels increased as pregnancy progressed and started to be statistically different from non pregnant values at gestational day 13 (P<0.05, Figure 4A). Although serum levels of CRP showed a tendency to be higher in the mid-pregnant than in the virgin group (P = 0.08, Figure 4B), a significant stimulatory effect was only evident at gestational day 18 (P<0.01).

### Il6 mRNA expression increased in WAT while Il6 and Il6ra mRNA decreased in the hypothalamus of pregnant WT mice

**Figure 5:**
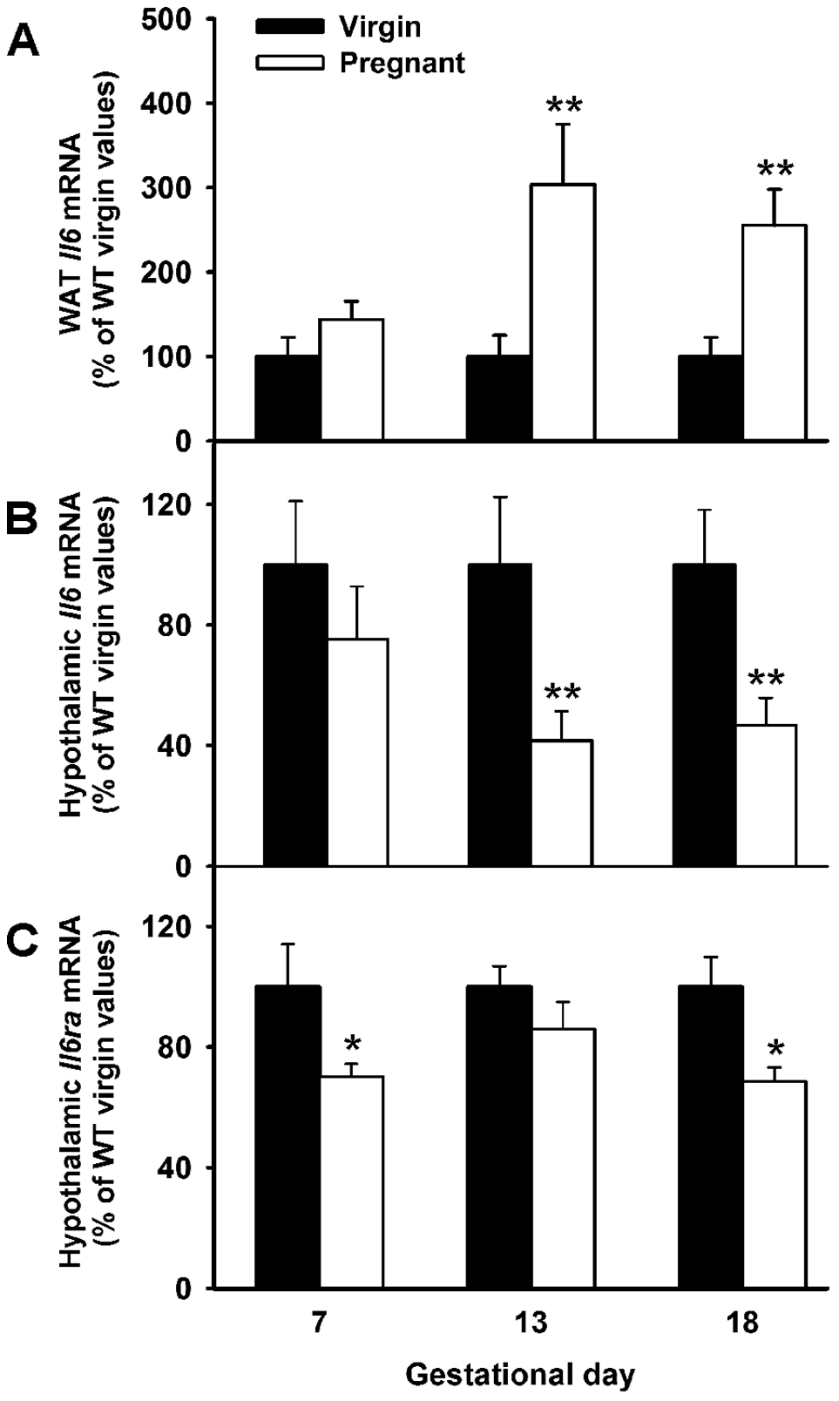
Reduced hypothalamic *Il6* and *Il6ra* but increased WAT *Il6* mRNA expression in pregnant WT mice. 12 weeks old WT (C57BL6) mice were time-pregnant and samples for RNA extractions were obtained from WAT and hypothalamus at early, mid and late pregnancy: gestational days 7 (n = 5), 13 (n = 8) and 18 (n = 6), respectively. Age matched virgin females were used as controls (n = 5–9). **A–C**. Gestational expression pattern of *Il6* mRNA in adipose tissue (A) and hypothalamus (B), and hypothalamic *Il6ra* mRNA levels (C) were determined by RT-qPCR. Expression of genes were measured in duplicates, normalized to *18s* and expressed in percentage to WT virgin control values. The bars represent the mean ± SEM. Two-tailed t-test, *P<0.05 and **P<0.01 versus corresponding WT virgin controls.

Adipose tissue *Il6* mRNA expression increased as pregnancy progressed. *Il6* mRNA levels were similar in adipose tissue of virgin and 7 days pregnant WT mice, but increased three fold on gestational days 13 and 18 (Figure 5A, percentage of WT virgin values at gestational days 13 and 18: 303.71±71.20 and 255.32±42,39, P<0.01, t-test). Conversely, hypothalamic *Il6* mRNA levels were a 60 and 50% lower in mid- and late pregnant mice than in corresponding virgin controls (Figure 5B, P<0.01). Hypothalamic *Il6ra* mRNA showed a slightly different profile during pregnancy from that of *Il6*, with decreased expression levels in pregnant mice at gestational days 7 and 18 in comparison to non-pregnant values (P<0.05, Figure 5C).

**Figure 6:**
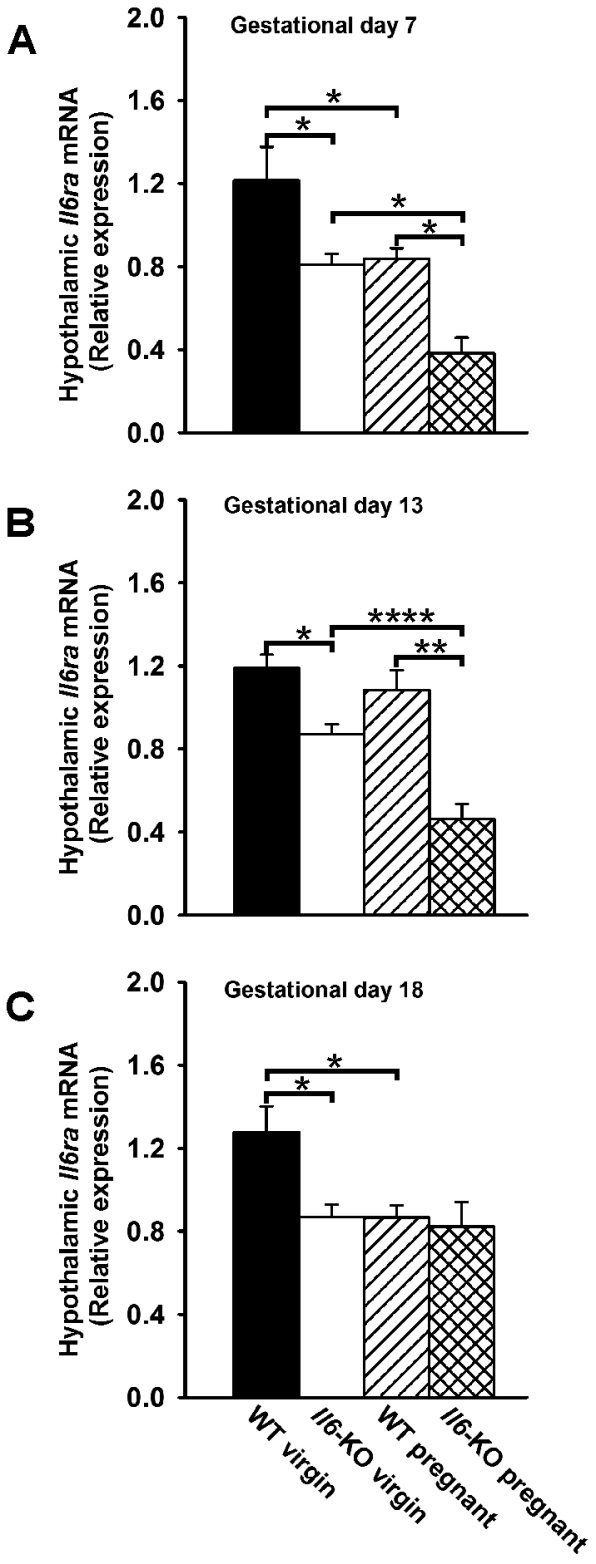
*Il6*-KO decreases hypothalamic *Il6ra* mRNA content in early, mid but not in late pregnant mice. 12 weeks old WT and *Il6*-KO mice were time-pregnant and RNA was harvested from hypothalamus of individual mice at early (n = 5), mid (n = 7–8) and late (n = 6–8). Age and genotype matched virgin females were used as controls (n = 5–9). **A–C**. *Il6ra* mRNA levels were measured in duplicates, normalized to *18s* and expressed as mean ± SEM. One-way ANOVA, *P<0.05, **P<0.01 and ****P <0.0001.

Recent published results [47] indicate that CNS (astrocyte-specific) deletion of *Il6* in female mice reduces IL6 receptor immunoreactivity in the cerebellum, but not in other brain areas such as hippocampus and cortex. Therefore, in order to confirm whether the observed reduction in hypothalamic *Il6ra* expression could be mediated by IL6 (whether of central or peripheral origin), the effect of total *Il6* deficiency on hypothalamic *Il6ra* mRNA levels at gestational days 7, 13 and 18 was also assessed (Figure 6A, B and C). Lack of *Il6* caused a significant inhibition on hypothalamic *Il6ra* mRNA content in virgin, early and mid-pregnant (p<0.05, P<0.05 and P<0.01, respectively, one-way ANOVA), but not in late pregnant mice (Figure 6C). These results suggest that at least in the non-pregnant state and at early and mid pregnancy, IL6 exerts a stimulatory effect on the hypothalamic expression of its receptor. However, the similar hypothalamic *Il6ra* mRNA content in late pregnant WT and *Il6*-KO mice probably reflects a compensatory mechanism during this stage of pregnancy.

### Npy, Agrp and Pomc mRNA expression in the ARC are up-regulated in Il6 deficiency during late-pregnancy

**Figure 7:**
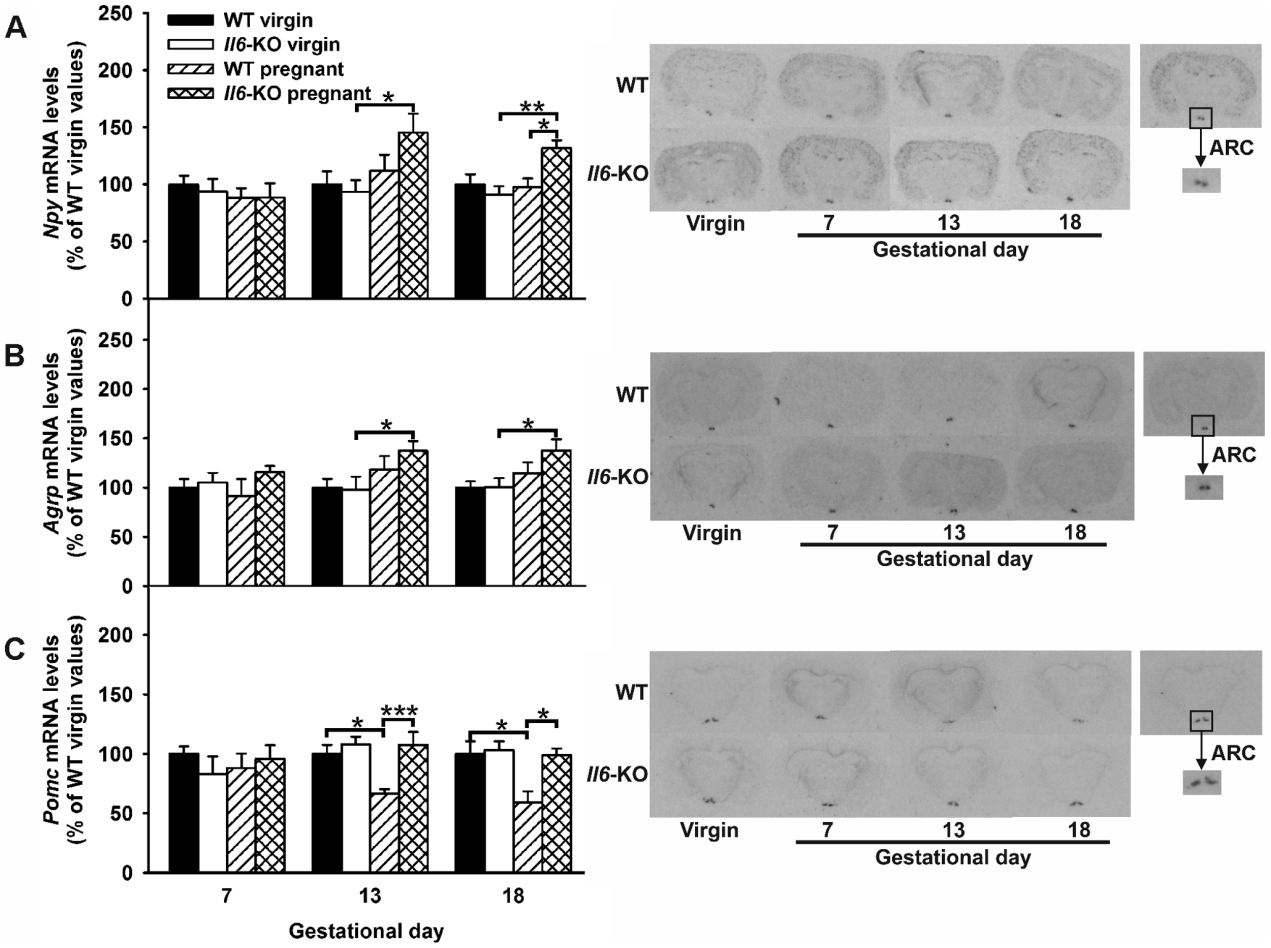
Increased ARC *Npy/Agrp* and unchanged *Pomc* mRNA expression in mid and late-pregnant *Il6*-KO mice. 12 weeks old WT and *Il6*-KO mice were time-pregnant and ARC gene expression was assessed by radioactive in situ hybridization in coronal brain sections from early (n = 6–8), mid (n = 6–9) and late (n = 12–16) pregnant mice. Age and genotype matched virgin females were used as controls (n = 5–16). **A–C**. *Npy* (A), *Agrp* (B) and *Pomc* (C) mRNA levels were normalized in percentage to WT virgin control values and expressed as mean ± SEM (right panel). Representative i*n situ* hybridization autoradiographic images are shown in the left panel. One-way ANOVA, *P<0.05, **P<0.01 and ***P <0.001.

In the pregnant rat, food intake and adiposity increases as a result of the resetting of central appetite control mechanisms at the level of the ARC, which leads to increased *Npy*/*Agrp* and decreased *Pomc* mRNA levels [31]. Thus, the effect of *Il6* deficiency on *Npy*/*Agrp* and *Pomc* gene expression during pregnancy was assessed by *in situ* hybridization. Nevertheless, our data showed that the *Npy* and *Agrp* expression in the ARC were not altered during normal pregnancy in the mouse (Figure 7A and B), whereas hypothalamic *Pomc* mRNA content in mid- and late pregnant mice were lower than in virgin animals (−37% as a mean, P<0.05, one-way ANOVA) (Figure 7C). As expected [17], the mRNA levels of all neuropeptides were unchanged by knockout of *Il6* in virgin female mice (Figure 7A, B and C). Instead, in mid and late pregnant animals, *Il6* deficiency caused a significant rise in *Npy/Agrp* expression as compared to non-pregnant mice and completely reverted the inhibitory effect of pregnancy on hypothalamic *Pomc* mRNA content (P<0.001 and P<0.05, for mid and late pregnant WT versus *Il6*-KO mice, respectively, one-way ANOVA).

### Lack of Il6 down-regulates Trh mRNA expression in the PVN during mid-pregnancy

**Figure 8:**
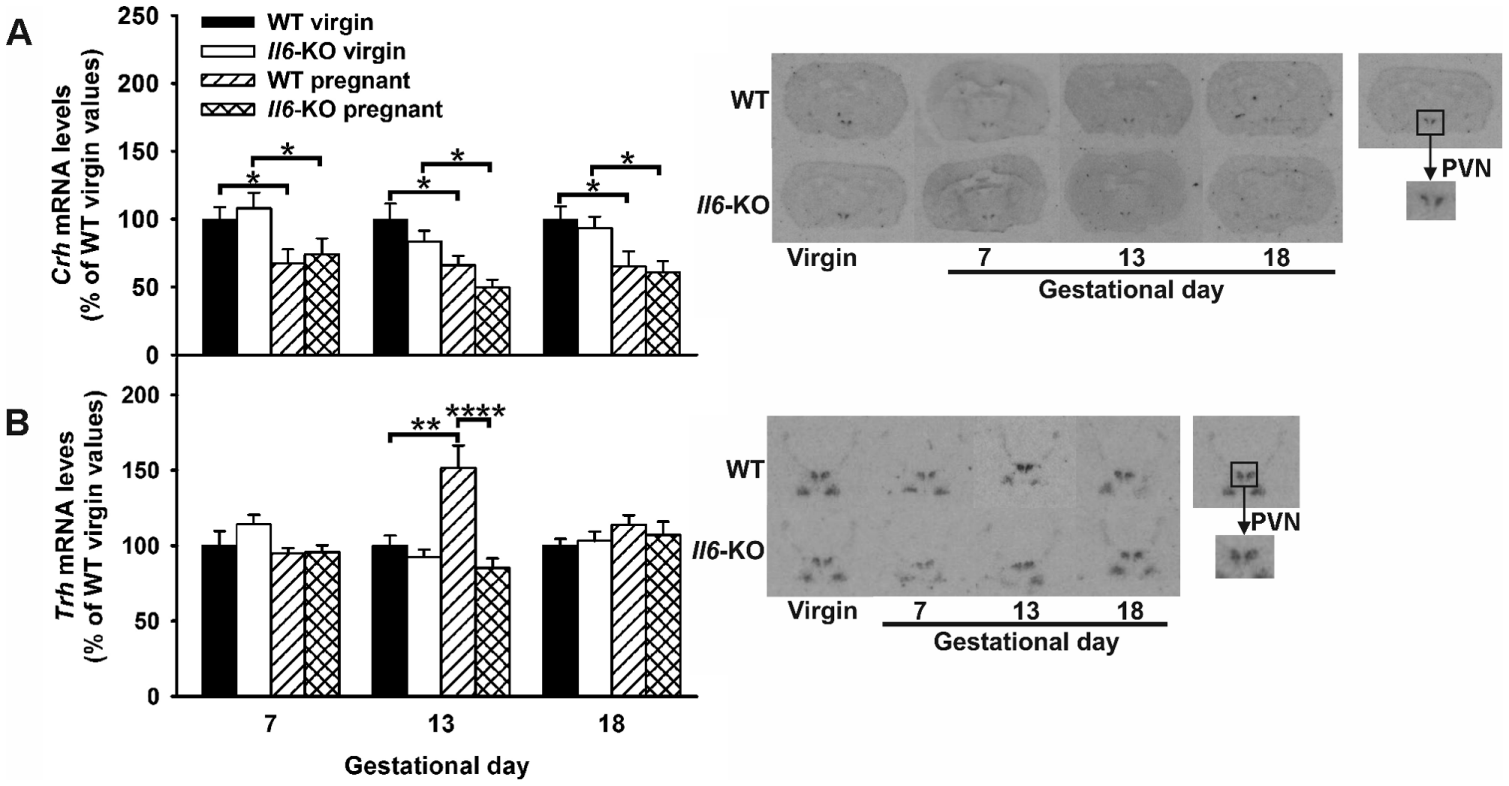
Increased PVN *Trh* mRNA content in mid-pregnant WT but not in *Il6*-KO mice. 12 weeks old WT and *Il6*-KO mice were time-pregnant and PVN gene expression was assessed by radioactive in situ hybridization in coronal brain sections from early (n = 8–10), mid (n = 7–12) and late (n = 12–15) pregnant mice. Age and genotype matched virgin females were used as controls (n = 7–15). **A–C**. *Crh* (A) and *Trh* (B) mRNA levels were normalized in percentage to WT virgin control values and expressed as mean ± SEM (right panel). Representative i*n situ* hybridization autoradiographic images are shown in the left panel. One-way ANOVA, *P<0.05, **P<0.01 and ****P <0.0001.

The aforementioned gestational changes in the hypothalamic peptidergic systems involved in the regulation of food intake and energy expenditure are not restricted to the ARC. In fact, the principal neuroendocrine outputs from the PVN, the parvicellular secretory neurons producing *Trh* and *Crh* are also affected [48]. Thus, the pattern of mRNA expression in the PVN of *Crh* and *Trh* was assessed during pregnancy, as were the influence of *Il6* deficiency on this setting. Our results showed that mRNA levels of *Crh* were decreased throughout gestation (−34% as a mean, P<0.05 for virgin versus pregnant WT mice at gestational days 7, 13 and 18, one-way ANOVA) (Figure 8A). This inhibition was similar in all pregnancy stages, as were the *Crh* mRNA content in the PVN from WT and *Il6*-KO in the virgin and pregnant groups, respectively.

Finally, the PVN *pro-Trh* mRNA levels, which were unaffected during early and late pregnancy in WT mice, were significantly increased on gestational day 13 in comparison to virgin animals (P<0.01, one way ANOVA, Figure 8B). Though, the *Trh* transcriptional expression was not modulated by *Il6* deficiency in the non-pregnant state, lack of *Il6* completely blunted the mid-pregnancy up-regulation of *Trh* mRNA levels (P<0.0001 for 13 days-pregnant WT mice versus *Il6-*KO mice).

## Discussion

Pregnancy is an excellent physiological model for studying how the CNS integrates peripheral signals, conveying information about the short- and long-term energy metabolic status of the body such as leptin, insulin or cholecystokinin, to alter hypothalamic function regulating food intake/energy balance and reproduction [49]. We report herein that IL6, an adipokine produced by the expanding adipose tissue, the hypothalamus and perhaps from other maternal sources like the placenta [50], [51], contributes to the neuroendocrine adaptation that occur in the dam's brain during this physiological demanding time.

Several studies point to a role of IL6 system as a key pathway involved in the central regulation of energy balance. Centrally but not peripherally administered IL6 reduces adiposity mainly by increasing energy expenditure [14] and thermogenesis [52], and to a lesser extent by decreasing food intake [23]. To address the possible role of IL6 in the central mechanisms associated to maternal hyperphagia and increased adiposity during pregnancy, in the present study we have used *Il6*-KO mice as a model. Therefore, young (12–15 weeks old) pregnant WT and *Il6*-KO mice were followed throughout gestation and their body weight, body composition and food intake was compared to those of non pregnant animals of both genotypes. As previously reported [26], we observed that *Il6*-KO female mice had lower body weight than WT mice due to a decrease in fat but not in lean body mass, body length or relative food intake. However, during pregnancy *Il6*-KO mice showed higher fat accretion than their WT controls, but similar fat mass and leptin levels prior parturition. This effect is not mediated by an increase in food intake in *Il6*-KO pregnant mice, whose food consumption in a weight corrected basis was similar to that of pregnant WT mice. Hence, taking into account the slight effect of *Il6* deficiency on maternal fat deposition observed in this study, it could be considered that IL6 is not of importance in the regulation of adiposity during this physiological setting. However, another alternative interpretation maybe that the increased fat mass gain in pregnant *Il6*-KO mice would be the end result of subtle mismatches between energy intake and the different components of energy expenditure.

Primary components of daily total energy expenditure include factors such as basal metabolic rate as well as the specific energy invested by animals on nutrient absorption/processing, diet-induced thermogenesis, non-shivering thermogenesis, and locomotor activity [53]. Small differences in these parameters between control and mutant mice, only evident after challenging conditions such as high fat diet feeding or cold exposure, might contribute under basal conditions to cumulative increments in fat mass and eventually to obesity [25], [41]. Detailed observation of the *Il6*-KO mice phenotype at young and late ages has been performed in several previous studies. Though *Il6*-KO mice may exhibit a lean phenotype at young age [26], it has been reported that *Il6* deficiency leads to obesity in the maturity without affecting feeding behaviour [14], [26], [54]. Faldt et al [26] but also Wernstedt et al [13] observed that pre-obese *Il6* deficient mice had a higher RER than their WT controls, indicating a preferential oxidation of carbohydrates vs fat, which has been postulated as a mechanism behind the subsequent development of obesity in this mouse model. In agreement with these data, our young *Il6*-KO female mice do exhibit an increased RER, which could justify at least in part its higher fat accretion during pregnancy. Additional mechanisms might include a decrease in energy expenditure in pregnant *Il6* deficient mice in comparison to WT controls as a result of an altered BAT functionality. This option seems plausible in light of the decreased UCP3 levels and the accumulation of lipid droplets observed in the BAT of *Il6*-KO mice at mid pregnancy, which suggests a reduction in the BAT thermogenic program in our model [55].

Central acting IL6 has been shown to reduce fat mass by altering the expression of key hypothalamic factors which control food intake and energy metabolism [24], [27], [54], [56]. To do so, IL6 binds to its specific cell surface receptor IL6ra which has been found to be largely expressed in well-known hypothalamic centers for energy-balance regulation such as the PVN [27] and the ARC [54]. A novel finding of this study was that *Il6* and *Il6*ra mRNA levels were down-regulated in the mouse hypothalamus during pregnancy. Central *Il6ra* gene expression is known to be activated in response to systemic immune challenges such as intravenous injections of lipopolysaccharide (LPS) or the pro-inflammatory cytokine interleukin-1 (IL1), but also in response to IL6 itself [57], [58]. In addition to its peripheral production, IL6 is also synthesized in the nervous system with cellular sources being neurons, astrocytes and microglia, which are recruited to produced this cytokine in response to different immunogenic stimulus including LPS endotoxemia [57], but also in response to overnutrition and obesity [56]. Whether IL6 of both origins plays distinct or parallel functions in a coordinate manner during chronic or acute inflammatory conditions has yet to be established. Whatever is the case, an stimulatory action of centrally produced IL6 on its own receptor is supported by the fact that acute i.c.v. IL6 treatment [59], but also astrocyte-specific knockout of *Il6* in female mice [47] has been shown to up-regulate and down-regulate brain *Il6ra* mRNA and protein levels. Considering the above mentioned results, a similar gestational pattern of hypothalamic expression might have been expected for both the ligand and its receptor. However, our data indicate that in the hypothalamus of early pregnant mice a reduction of *Il6ra* mRNA levels occurs without changes in central *Il6* expression and circulating levels. Furthermore, we also report that lack of the ligand in non-pregnant and early pregnant animals further down-regulates the hypothalamic expression of *Il6ra*. The reason for this discrepancy is unknown, but it points to the involvement of other factors as possible modulators of the *Il6ra* gene transcription during this stage of pregnancy.

At mid-pregnancy *Il6ra* mRNA levels returned to non pregnant values while *Il6* gene expression was already reduced. At this gestational time increased peripheral production of IL6, sourced from the adipose tissue, as we demonstrate here, and from a yet fully functional placenta [51], [60], might contribute to the high circulating concentrations of this cytokine in mid-pregnant mice. These results are in agreement with previous reports showing that serum IL6 are elevated as early as gestational day 11 in the mouse [37]. Since we did not measure IL6 CSF levels due to methodological constrains (low sample volume yields for available ELISA assays, i.e. only 5 µl/per animal [61]), whether blood-borne IL6 is able to reach the brain parenchyma and effectively restore the hypothalamic expression of its receptor remains to be elucidated. However, in accordance to a stimulatory effect of IL6 (whether of central or peripheral origin) on hypothalamic *Il6ra* mRNA levels, we report that lack of *Il6* in mid-pregnant animals markedly diminishes its receptor gene expression. In turn, we provide evidences that at late pregnancy a peripheral low-grade inflammatory condition, as measured by elevated serum IL6 and CRP concentrations, is associated with reduced hypothalamic *Il6* and *Il6ra* mRNA levels. Perhaps, these results reflect a decreased passage of IL6 trough the blood brain barrier, a fact similar to that described for leptin in late pregnant rodents [28], [31], [33], [35], [62], whose central nervous system become “ leptin resistant ” at the end of pregnancy when leptinemia increases – thus avoiding the suppressive effects of leptin on food intake and fat accumulation. Therefore, the combined effect of a decreased entrance of IL6 into the CNS and a decreased hypothalamic *Il6* gene expression might contribute to reduce *Il6ra* mRNA levels. Surprisingly, knockout of *Il6* in late pregnant animals did not affect the hypothalamic expression of its receptor, probably reflecting a local compensatory mechanism exerted by other cytokines. In support of this hypothesis, serum levels of CRP, a systemic marker of inflammation, did not differ between late pregnant WT and *Il6*-KO mice and it has been recently demonstrated that *Il1-beta* expression is increased in hypothalamus of obese *Il6*-KO mice [42], [54].

*Il6ra* is expressed in a number of hypothalamic nuclei involved in the regulation of energy balance where, as mentioned before, it mediates the effects of this cytokine on energy expenditure and thermogenesis. However, the experimental approach used in the current study was designed to determine global changes in *Il6ra* expression in the pregnant mouse hypothalamus, and not to establish the contribution from individual neuronal populations. Therefore, in an attempt to further dissect this issue, we analyzed in our model the transcriptional activity of the main peptidergic systems involved in this function within the ARC and PVN.

The ARC in particular, where *Il6ra* is widely expressed, it is considered as a key integrative center of internal signals encoding energy status through NPY/AGRP and POMC neurons [7], [56]. The results in the present study revealed a differential transcriptional modulation of these genes in the mouse ARC during mid to late pregnancy from that previously reported in the rat [28], [31], [38], probably reflecting a biological difference between species. Thus, in WT mice the exponential rise in food intake rate observed from gestational days 13 to 18 seems to be driven by a reduction in *Pomc* mRNA levels, while *Npy*/*Agrp* expression remains unchanged. A similar absence of gestational up-regulation of *Npy* and *Agrp* production in the mouse ARC has been previously reported by some authors [6], [63] but not by others [64], the reason for this discrepancy is unknown but it could be related to the different methodologies employed (in situ hybridization versus real time-PCR). Lack of *Il6* in mice restored *Pomc* mRNA content during mid- to late pregnancy and increased hypothalamic *Agrp* and *Npy* mRNA levels. These data suggest that in absence of IL6 signaling during latest stages of pregnancy a compensatory mechanism takes place on POMC and NPY/AGRP neurons, so far allowing maternal hyperphagia and increased adiposity to be maintained. In fact, results from a recent report demonstrated that knockout of *Il6* in male mice exerts a similar stimulatory action at the level of the NPY/AGRP neurons in the ARC after 4 weeks of cold exposure, i.e. after another extremely energetic challenging condition [54]. Conversely, exercise-induced hypothalamic IL6 and IL10 in obese rats [56] and *Il6* astrocyte-targeted overexpression in female mice have been shown to reduce *Npy/Agrp* mRNA levels and increase *Pomc* expression in the ARC [24], [65].

Another novel finding in the current study was that, in the pregnant mouse, expression of *Trh* in the PVN was up-regulated during mid-pregnancy and this positive effect was blunted by knockout of *Il6*. The PVN acts as a primary integrative center for peripheral cytokine signaling to CNS and stimulation of a variety of physiological, neuroendocrine and behavioral CNS responses including: suppression of food intake, thermogenesis and HPA axis activation [7]. However, these results were unexpected taking into account that hypothalamic *Pomc* mRNA content was depressed at this gestational time point and TRH neurons in the PVN are densely innervated by alpha-MSH-producing neurons, which potently stimulates *Trh* expression and secretion [8]. The functional relevance of the *Trh* transcriptional modulation during mid-pregnancy remains to be determined but, considering the important role of this neuropeptide in the regulation of energy homeostasis and its central effects on thermogenesis [8], a role as triggering signal of the increase in maternal basal metabolic rate seen in the latter stages of mouse pregnancy [66] might be hypothesized. The fact that lack of *Il6* in midpregnant mice reduced the PVN *Trh* mRNA content to non-pregnant levels was equally unexpected. Despite the high level of co-expression of *Trh* and *Il6ra* in the mouse hypothalamus [27], published data do not support a role of IL6 as a direct modulator of *Trh* gene transcription [67]. Nevertheless, other pro-inflammatory cytokines such as TNF alpha and IL1 have been shown to affect the HPT axis at multiple levels leading to marked decreases in hypothalamic TRH expression [68]. Whether a compensatory action of this cytokines on TRH mRNA levels is established in the hypothalamus of *Il6*-KO pregnant mice will merit further investigation.

A final issue to take into account when considering the findings of the present study is the possible source of experimental variability induced by the homozygous breeding scheme used to generate the experimental animals. Due to experimental constrains and to ensure genetic homogeneity, we have employed as breeding founders animals from a C57BL6J-congenic line of IL-6 deficient mice and its corresponding controls from the recipient strain, all sourced from the same vendor. Although, this breeding design is being largely used in many studies, it has a potential-limitation of not taking into account the possible impact of maternal/paternal genotype, *in utero* environment and maternal nursing on the metabolism of the offspring [69].

In summary, we show that pregnancy in the mouse is associated with a progressive increase in circulating IL6 levels, while hypothalamic *Il6* and *Il6ra* expression are depressed. This effect might contribute to a decreased sensitivity to the catabolic action of IL6 during this physiological state, as shown by increase fat accretion in *Il6*-KO pregnant mice. Lack of *Il6* in mice differentially modulates the gestational transcriptional profile of energy balance regulating peptides at the level of the ARC and PVN, with mayor stimulatory and inhibitory effects on *Agrp*, *Npy* and *Pomc* as well as *Trh* gene expression during mid- and late-pregnancy. Collectively, the results of the present study suggest a role of IL6 in the central homeostatic mechanisms that regulate body fat during pregnancy.

## Supporting Information

Figure S1 **Lean body mass content in WT and** ***Il6*** **-KO mice during pregnancy.** Longitudinal measurements of body composition were performed in 15 weeks old mice at gestational days 7, 13 and 18, age and genotype matched virgin females were used as controls (virgin, n = 5–7 and pregnant n = 9–10/animals per group). **A–B**. Lean body mass of virgin and pregnant WT and Il6-KO mice at the beginning (gestational day 7) and the end of the experimental period (gestational day 18) as expressed in an absolute (A) or a relative to weight basis (B). Two-way ANOVA for repeated measurements, **P<0.01.
(TIF)

Figure S2 **Subcutaneous fat mass content in WT and** ***Il6*** **-KO mice during pregnancy.** For transversal analysis of absolute (right panel) and relative dissected subcutaneous fat mass (middle and left panel) independent groups of 12 weeks old time-pregnant mice were sacrificed on gestational days 7 (A, n = 8–9), 13 (B, n = 8–9) and 18 (C, n = 11). Age and genotype matched virgin females were used as controls (n = 7–11). Relative fat mass values were calculated in percentage to maternal body weight (middle panel), excluding the contribution of placentae and fetuses, and normalized to virgin control values of each genotype (C). Data are expressed as mean ± SEM. One-way ANOVA, *P<0.05, **P<0.01, ***P <0.001 and ****P<0.0001; two-tailed t-test #P<0.05 versus corresponding WT pregnant controls.
(TIF)

Figure S3 **Decreased serum leptin levels in virgin but not in late pregnant Il6-KO mice.** Circulatin leptin levels were assessed in 12 weeks old time-pregnant mice (n = 10/group). Age and genotype matched virgin females were used as controls (n = 10). Data are expressed as mean ± SEM. One-way ANOVA, *P<0.05 and ***P <0.001.
(TIF)

Figure S4 **BAT of mid-pregnant** ***Il6*** **-KO mice show features of an altered thermogenic program.** **A–B**. *Ucp1* (A) and *Ucp3* (B) mRNA levels in BAT samples from mid-pregnant WT and *Il6*-KO mice as determined by RT-qPCR (n = 7–8). Age and genotype matched virgin females were used as controls (n = 7–8). **C–D**. Protein levels of both thermogenic markers (C, UCP1 and D, UCP3) were also assessed by western-blot (n = 6) and representative images are shown in the right panels. Data are expressed as mean ± SEM. One-way ANOVA, *P<0.05. **E**. Representative pictures (10× magnification) of BAT samples from mid-pregnant WT and *Il6*-KO mice stained with oil-red to determine the accumulation of neutral fat.
(TIF)

Table S1 **Primers and probes used for real-time PCR and in situ hybridization.**
(DOCX)

Table S2 **Litter size and weight of 18 days-conceptuses and new-born mice.**
(DOCX)
