# Supplementary figures and images for: Interleukin 6 Deficiency Modulates the Hypothalamic Expression of Energy Balance Regulating Peptides during Pregnancy in Mice
Source: PLoS One. 2013 Aug 28;8(8):e72339. doi: 10.1371/journal.pone.0072339 (PMC3756067; doi:10.1371/journal.pone.0072339)

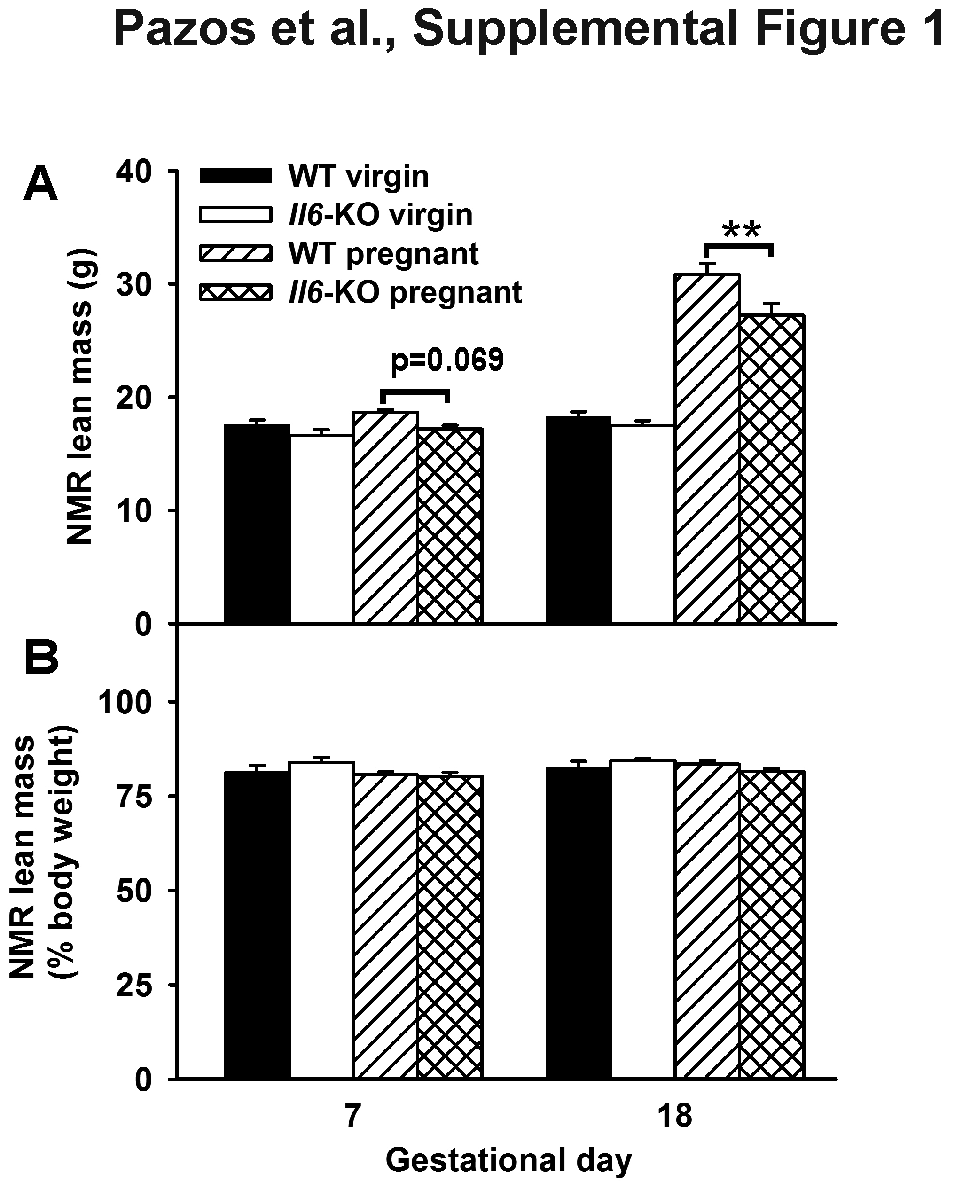

Supplement: Figure S1 — Lean body mass content in WT and Il6 -KO mice during pregnancy. Longitudinal measurements of body composition were performed in 15 weeks old mice at gestational days 7, 13 and 18, age and genotype matched virgin females were used as controls (virgin, n = 5–7 and pregnant n = 9–10/animals per group). A–B. Lean body mass of virgin and pregnant WT and Il6-KO mice at the beginning (gestational day 7) and the end of the experimental period (gestational day 18) as expressed in an absolute (A) or a relative to weight basis (B). Two-way ANOVA for repeated measurements, **P<0.01. (TIF) [file pone.0072339.s001.tif]

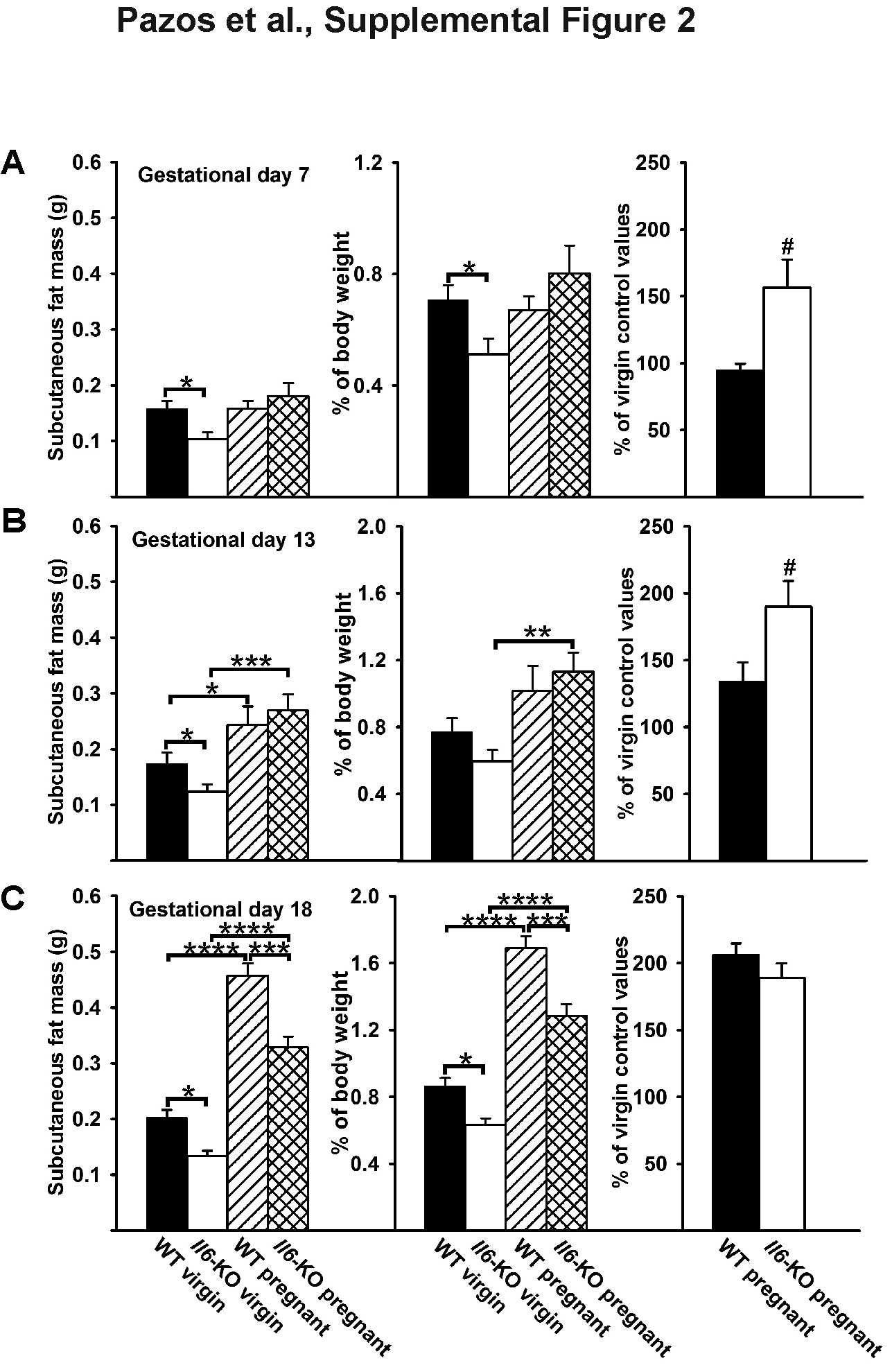

Supplement: Figure S2 — Subcutaneous fat mass content in WT and Il6 -KO mice during pregnancy. For transversal analysis of absolute (right panel) and relative dissected subcutaneous fat mass (middle and left panel) independent groups of 12 weeks old time-pregnant mice were sacrificed on gestational days 7 (A, n = 8–9), 13 (B, n = 8–9) and 18 (C, n = 11). Age and genotype matched virgin females were used as controls (n = 7–11). Relative fat mass values were calculated in percentage to maternal body weight (middle panel), excluding the contribution of placentae and fetuses, and normalized to virgin control values of each genotype (C). Data are expressed as mean ± SEM. One-way ANOVA, *P<0.05, **P<0.01, ***P <0.001 and ****P<0.0001; two-tailed t-test #P<0.05 versus corresponding WT pregnant controls. (TIF) [file pone.0072339.s002.tif]

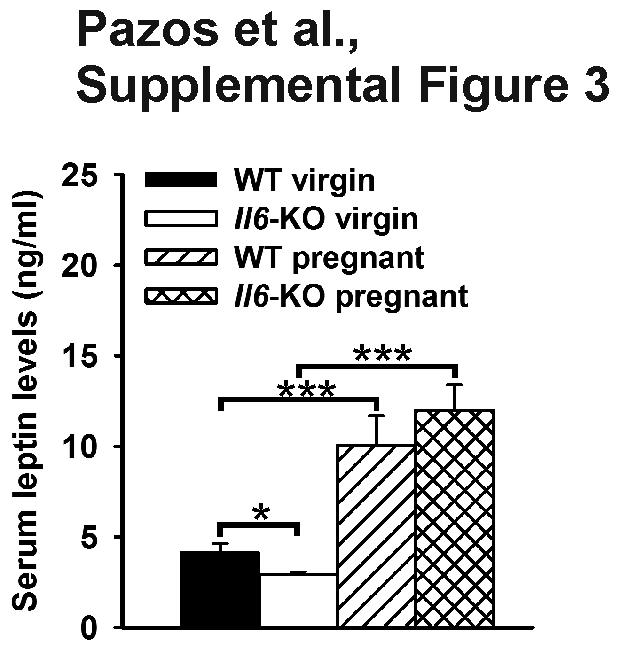

Supplement: Figure S3 — Decreased serum leptin levels in virgin but not in late pregnant Il6-KO mice. Circulatin leptin levels were assessed in 12 weeks old time-pregnant mice (n = 10/group). Age and genotype matched virgin females were used as controls (n = 10). Data are expressed as mean ± SEM. One-way ANOVA, *P<0.05 and ***P <0.001. (TIF) [file pone.0072339.s003.tif]

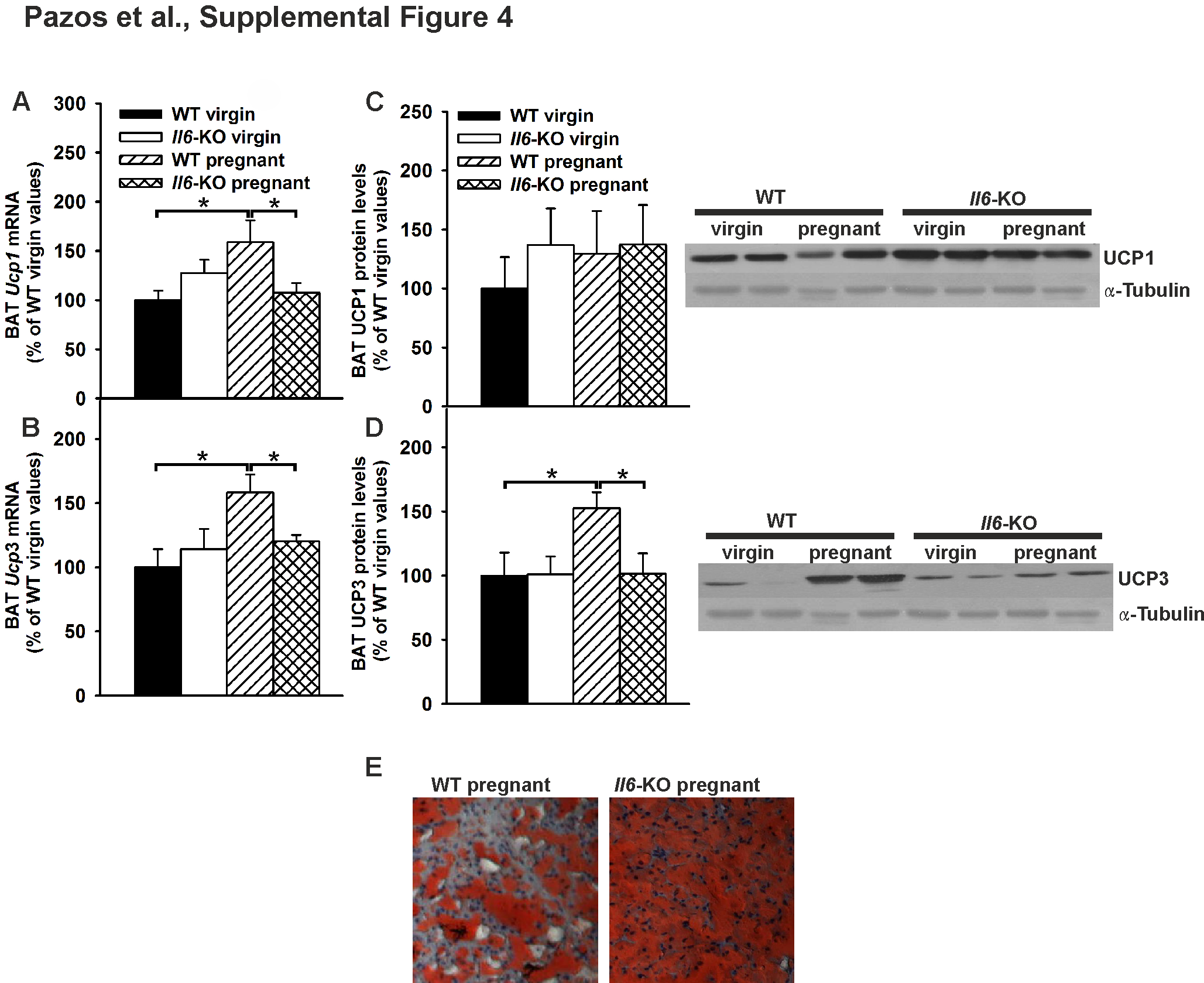

Supplement: Figure S4 — BAT of mid-pregnant Il6 -KO mice show features of an altered thermogenic program. A–B. Ucp1 (A) and Ucp3 (B) mRNA levels in BAT samples from mid-pregnant WT and Il6-KO mice as determined by RT-qPCR (n = 7–8). Age and genotype matched virgin females were used as controls (n = 7–8). C–D. Protein levels of both thermogenic markers (C, UCP1 and D, UCP3) were also assessed by western-blot (n = 6) and representative images are shown in the right panels. Data are expressed as mean ± SEM. One-way ANOVA, *P<0.05. E. Representative pictures (10× magnification) of BAT samples from mid-pregnant WT and Il6-KO mice stained with oil-red to determine the accumulation of neutral fat. (TIF) [file pone.0072339.s004.tif]
