# Supplementary material for: Interleukin 6 Deficiency Modulates the Hypothalamic Expression of Energy Balance Regulating Peptides during Pregnancy in Mice
Source: PLoS One. 2013 Aug 28;8(8):e72339. doi: 10.1371/journal.pone.0072339 (PMC3756067; doi:10.1371/journal.pone.0072339)
Supplement: Table S1 — Primers and probes used for real-time PCR and in situ hybridization. (DOCX) [file pone.0072339.s005.docx]

**Table S1. Primers and probes used for real-time PCR and in situ hybridization.**

| **Gene** | **Gene bank** | ***Primers and* probes sequences^a^** | **Product size (bp)/sequence^d^** | **Modified from^c^** |
| --- | --- | --- | --- | --- |
| *Rn18s* | NR_003278.1 | F:5´CGCCGCTAGAGGTGAAATTC3´ | 101 | 1 |
|  |  | R:5´CGAACCTCCGACTTTCGTTCT3´ |  |  |
|  |  | Pb:5´CCGGCGCAAGACGGACCAGA3´ |  |  |
| *Il6* | NM_031168.1 | F:5´CTATACCACTTCACAAGTCGGAGG3´ | 76 | 2 |
|  |  | R:5´TGCACAACTCTTTTCTCATTTCC3´ |  |  |
|  |  | Pb:5´TTAATTACACATGTTCTCTGGGAAATCG3´ |  |  |
| *Il6ra* | NM_010559.2 | Mm00439653_m1^b^ | 98 | - |
| *Ucp1* | NM_009463.2 | F:5´GCAGATATCATCACCTTCCCG3´  R: 5´CCTGGCCTTCACCTTGGAT3´  Pb: 5´TGGACACTGCCAAAGTCCGCCTTC 3´ | 66 | 3 |
| *Ucp3* | N M _009464.3 | F:5´GAAGATGGTGGCTCAGGAGG3´  R: 5´AAGCTCCCAGACGCAGAAAG3  Pb: 5´CCACGGCCTTCTACAAAGGATTTGTGC3´ | 73 | 3 |
| *Npy* | NM_023456.2 | 5´GAGCGGAGTAGTATCTGGCCATGT  CCTCTGCTGGCGCGTCCT3´ | 186-227 | 4 |
| *Agrp* 1 | NM_007427.2 | 5´TGCAGCAGAACTTCTTCTGCTCGG  TCTGCAGTTGTCTTCTTGAGG3´ | 354-398 | 4 |
| *Agrp* 2 | NM_007427.2 | 5´TGCTTGCGGCAGTAGCAAAAGGCA  TTGAAGAAGCGGCAGTAGCA3´ | 526-569 | 4 |
| *Pomc* | NM_008895.3 | 5´CGTTCTTGATGATGGCGTTCTTGA  AGAGCGTCACCAGGGGCGTCT3´ | 793-837 | 4 |
| *Crh* | NM_205769.1 | 5´CAGTTTCCTGTTGCTGTGAGCTTG  CTGAGCTAACTGCTCTGCCC3´ | 677-720 | 5 |
| *Trh* | NM_009426.2 | 5´GTACAGAGGGCCCAGGGTTAGCA  TCTAAGAAGAAAACCAGTG3´ | 943-984 | 6 |

^a^F, Forward; R, reverse; Pb, probe

^b^Gene Expression Assay Applied Biosystems

^c^Please see supplemental references

^d^Complementary mRNA sequence

**Supplemental references for Table S1 [**[**1-6**](#_ENREF_1)**]**

**References**

1. Capurso G, Lattimore S, Crnogorac-Jurcevic T, Panzuto F, Milione M, et al. (2006) Gene expression profiles of progressive pancreatic endocrine tumours and their liver metastases reveal potential novel markers and therapeutic targets. Endocr Relat Cancer 13: 541-558.

2. Rioja I, Bush KA, Buckton JB, Dickson MC, Life PF (2004) Joint cytokine quantification in two rodent arthritis models: kinetics of expression, correlation of mRNA and protein levels and response to prednisolone treatment. Clin Exp Immunol 137: 65-73.

3. Mashiko S, Ishihara A, Iwaasa H, Sano H, Oda Z, et al. (2003) Characterization of neuropeptide Y (NPY) Y5 receptor-mediated obesity in mice: chronic intracerebroventricular infusion of D-Trp(34)NPY. Endocrinology 144: 1793-1801.

4. Wang H, Storlien LH, Huang XF (2002) Effects of dietary fat types on body fatness, leptin, and ARC leptin receptor, NPY, and AgRP mRNA expression. Am J Physiol Endocrinol Metab 282: E1352-1359.

5. Yamamoto Y, Ueta Y, Date Y, Nakazato M, Hara Y, et al. (1999) Down regulation of the prepro-orexin gene expression in genetically obese mice. Brain Res Mol Brain Res 65: 14-22.

6. Broberger C (1999) Hypothalamic cocaine- and amphetamine-regulated transcript (CART) neurons: histochemical relationship to thyrotropin-releasing hormone, melanin-concentrating hormone, orexin/hypocretin and neuropeptide Y. Brain Res 848: 101-113.
