# Supplementary material for: Interleukin 6 Deficiency Modulates the Hypothalamic Expression of Energy Balance Regulating Peptides during Pregnancy in Mice
Source: PLoS One. 2013 Aug 28;8(8):e72339. doi: 10.1371/journal.pone.0072339 (PMC3756067; doi:10.1371/journal.pone.0072339)
Supplement: Table S2 — Litter size and weight of 18 days-conceptuses and new-born mice. (DOCX) [file pone.0072339.s006.docx]

**Table S2. Litter size and weight of 18 days-conceptuses and new-born mice.**

|  | **Gestational day 18^a^** | | **Post-partum day 1^b^** | |
| --- | --- | --- | --- | --- |
| **Genotype** | **Litter size** | **Conceptus Weight (g)** | **Litter size** | **Weight (g)** |
| WT | 8.09±0.57 | 1.52±0.06 | 7.33±0.9 | 1.25±0.01 |
| *Il6*-KO | 8.01±0.46 | 1.57±0.04 | 7.32±0.8 | 1.28±0.01 |

^a^n=8 dams/group

^b^n=7-8 dams/group
